# Molybdenum Disulfide as Tunable Electrochemical and Optical Biosensing Platforms for Cancer Biomarker Detection: A Review

**DOI:** 10.3390/bios13090848

**Published:** 2023-08-25

**Authors:** Ziyue Qin, Jiawei Zhang, Shuang Li

**Affiliations:** 1 Medical College, Tianjin University, Tianjin 300072, China; 2021235042@tju.edu.cn (Z.Q.); jiawei_zhang@tju.edu.cn (J.Z.); 2 Academy of Medical Engineering and Translational Medicine, Tianjin University, Tianjin 300072, China

**Keywords:** molybdenum disulfide, electrochemical sensor, optical sensor, cancer biomarkers, detection

## Abstract

Cancer is a common illness with a high mortality. Compared with traditional technologies, biomarker detection, with its low cost and simple operation, has a higher sensitivity and faster speed in the early screening and prognosis of cancer. Therefore, extensive research has focused on the development of biosensors and the construction of sensing interfaces. Molybdenum disulfide (MoS_2_) is a promising two-dimensional (2D) nanomaterial, whose unique adjustable bandgap shows excellent electronic and optical properties in the construction of biosensor interfaces. It not only has the advantages of a high catalytic activity and low manufacturing costs, but it can also further expand the application of hybrid structures through different functionalization, and it is widely used in various biosensors fields. Herein, we provide a detailed introduction to the structure and synthesis methods of MoS_2_, and explore the unique properties and advantages/disadvantages exhibited by different structures. Specifically, we focus on the excellent properties and application performance of MoS_2_ and its composite structures, and discuss the widespread application of MoS_2_ in cancer biomarkers detection from both electrochemical and optical dimensions. Additionally, with the cross development of emerging technologies, we have also expanded the application of other emerging sensors based on MoS_2_ for early cancer diagnosis. Finally, we summarized the challenges and prospects of MoS_2_ in the synthesis, functionalization of composite groups, and applications, and provided some insights into the potential applications of these emerging nanomaterials in a wider range of fields.

## 1. Introduction

Cancer is the world’s leading cause of death and the second most common disease [1]. At present, more than 200 types of cancers have been found. In general, imaging technologies such as ultrasound, positron emission tomography (PET), magnetic resonance imaging (MRI), and computed tomography (CT) are used for early screening, followed by confirmation through tissue biopsy and histology, so that patients can be treated in a timely manner, which can dramatically reduce cancer mortality [2,3,4]. However, traditional cancer detection methods are often invasive, expensive, complex, and time consuming. Rapid diagnosis and early prevention are crucial for the clinical treatment and management of cancer [5]. Cancer biomarkers, as important components of detection, prognosis, and providing an etiological analysis of cancer, are abnormal quantities of biological molecules generated by the body’s response to the disease or directly by the cancer tumor itself, including DNA, RNA, genes, proteins, enzymes, peptides, exosomes, and metabolomics [6]. So far, the main cancer biomarkers that have been discovered include carcinoembryonic antigen (CEA), carbohydrate antigen 125 (CA125), carbohydrate antigen 15-3 (CA15-3), human epidermal growth factor receptor 2 (HER2), vascular endothelial growth factor 165 (VEGF_165_), tissue-specific antigen (TPS), prostate-specific antigen (PSA), alpha-fetoprotein (AFP), squamous cell carcinoma antigen (SCCA), circulating tumour cells (CTCs), microRNAs, and exosomes [7]. They typically exist in the blood, urine, tears, oral fluids, and other tissues [8]. Cancer biomarker detection has accelerated the process of cancer diagnosis, and can obtain higher sensitivity and faster cancer screening. Enzyme-linked immunosorbent assay (ELISA) [9], polymerase chain reaction (PCR) [10], clustered regularly interspaced short palindromic repeats Cas9 (CRISPR-Cas9) [11], loop-mediated isothermal amplification (LAMP), time-resolved fluorescence spectroscopy (TR-FS), radioimmunoassay (RIA), and electrophoresis [12] have been used for the detection of cancer biomarkers [13]. In addition, emerging technologies such as artificial intelligence, long read sequencing, microarrays, DNA methylation, and liquid biopsy are also committed to the development and high throughput profiling of many biomarkers to strengthen cancer management and improve early screening [14,15].

In recent years, compared with traditional technologies, biosensors have potential advantages such as a high sensitivity and selectivity, high accuracy, low cost, fast detection, high stability, availability, and ease of operation. They play an important role in diagnosing and quantitatively analysing biomarker concentrations, and are widely used in various fields such as healthcare, food inspection, and environmental testing [16]. Biosensors use various biomolecules as biometric recognition components, which are fixed on the sensor surface and converted into measurable electronic or optical signals through biological responses with the detection target substance for cancer biomarker detection [17]. Biosensors can be divided into electrochemical, optical, mass-dependent, and radiation sensitive biosensing platforms based on different transduction principles [18,19]. Developing efficient and practical biosensors usually requires consideration of the following aspects: (1) synthesis, manufacture, and assembly of suitable sensing materials; (2) selecting appropriate recognition or capture molecules; and the (3) integration of sensor surfaces with biomolecules [20,21]. With the development of nanotechnology in medicine and biotechnology, more and more researchers are combining different types of nanomaterials with optical, electrical, mechanical, and magnetic sensors to design nanosensors for the detection of cancer biomarkers [22]. Nanobiosensors are generally composed of nanomaterials and a sensor based on biometric recognition elements [23]. They can be combined according to the interaction of the affinity bond, covalent bond, cross-linking, capture, and physical adsorption [24].

Among the various nanomaterials, 2D-layered nanomaterials have attracted widespread research interest due to their quantum confinement, high absorption coefficient, high specific surface area, and tunable bandgap characteristics [25]. Among them, graphene has excellent physical properties, chemical adjustability, and application potential, and its synthesis, properties, and applications are widely known [26]. The impressive performance of graphene in various fields has aroused strong interest in the exploration of a wider range of 2D-layered nanomaterials “beyond graphene” [27]. Transition metal dichalcogenides (TMDs), as a new class of stable inorganic graphene analogues, have been further studied. Among them, MoS_2_ is regarded as a representative of TMDs. Its single molecular layer is composed of an atomic layer of transition metal Mo sandwiched between two sulfur elements S [28]. Mo atoms and S atoms are closely connected by forming a strong covalent bond through coordination, and the interlayer is connected by a weak van der Waals force. This weak connection mode between layers provides conditions for MoS_2_ stripping to form a single-layer 2D planar structure [29], showing unique electronic, optical, mechanical, and chemical properties [30,31,32]. Most importantly, due to the confinement of electrons/holes in ultra-thin planar structures, MoS_2_ is highly sensitive to changes in the microenvironment [33], thus exhibiting advantages in the construction of biosensing interfaces [34,35].

**Scheme 1:**
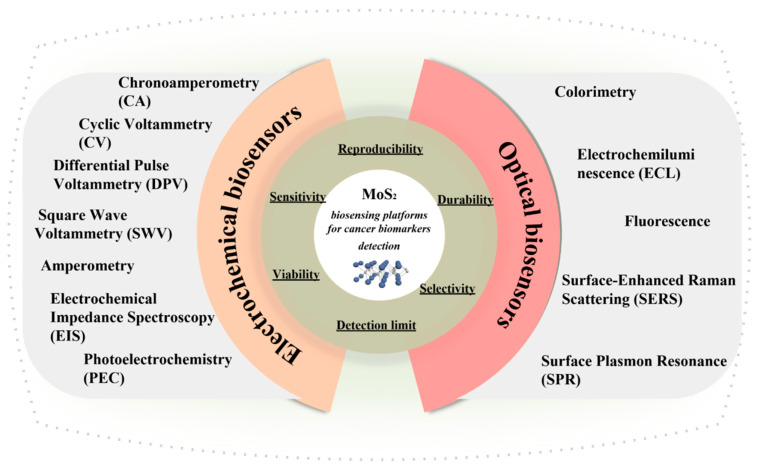
Electrochemical/optical sensing platforms based on MoS_2_ in cancer biomarker detection.

A large number of literature works have reported that different types of biosensing platforms based on MoS_2_ are used to detect various biomarkers, considering the needs of cancer biomarker detection in terms of high sensitivity, high reproducibility, easy processing, low cost, and miniaturization. In this work, we comprehensively reviewed and summarized the application of electrochemical/optical sensing platforms based on MoS_2_ in cancer biomarker detection, and highlighted the excellent characteristics and application performance of MoS_2_ and its composite structures (Scheme 1). Firstly, the research status of early cancer diagnosis and traditional/emerging biosensing technologies for cancer biomarkers detection were investigated, with a focus on introducing 2D nanomaterials represented by MoS_2_. Secondly, the structures and synthesis methods of MoS_2_ were discussed in detail, as well as the unique properties and advantages/disadvantages exhibited by different structures. Then, the focus was on summarizing the recent electrochemical and optical sensing work of MoS_2_ and its composite structures in the field of cancer biomarker detection. In addition, we expanded the application of other emerging sensors based on MoS_2_ for early cancer diagnosis. Finally, we summarized the challenges and prospects of MoS_2_ in terms of the synthesis, functionalization of composite groups, and applications, and provided some insights into the enormous potential of these emerging 2D nanomaterials in the field of cancer biomarkers detection.

## 2. Synthesis and Characteristics of Molybdenum Disulfide

**Figure 1:**
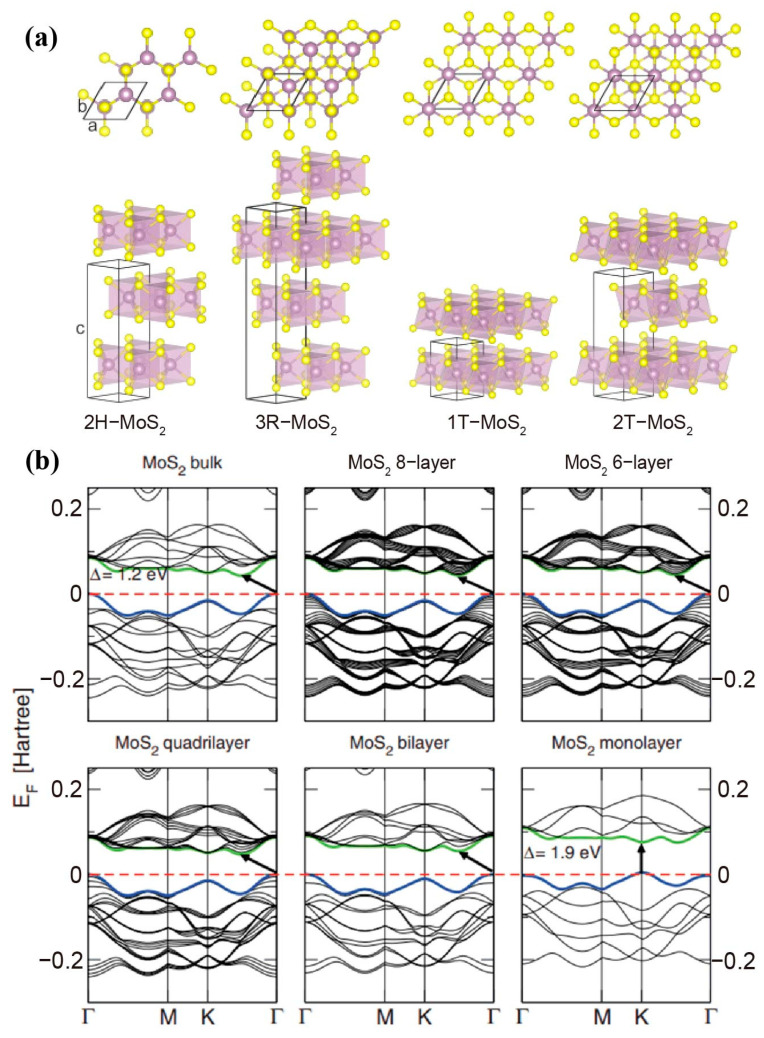
(**a**) Various crystal structures of MoS_2_ (1T, 2H, 3R, and 2T) [38]. Copyright 2014, Elsevier. (**b**) Electronic band structure diagram of MoS_2_ with different thicknesses [44]. Copyright 2011, American Physical Society.

MoS_2_ has a hexagonal lattice structure, with three common crystal structures of 1T, 2H, and 3R, and their corresponding point groups are D6d, D6h, and C3v [36]. In addition, an additional hypothetical 2T structure of MoS_2_ has been studied and reported [37]. Accordingly, 1, 2, and 3 represent the number of layers, T represents the triagonal configuration, H represents the hexagonal configuration, and R represents the rhombohedral configuration. As shown in Figure 1a, the MoS_2_ layers in the 2H, 3R, 1T, and additional 2T crystal structures are arranged in anti-parallel AB, parallel ABC, parallel AA, and anti-parallel AA’ stacking sequences, respectively [38]. The properties of MoS_2_ may show different characteristics according to the change in crystal structure [39]. Among these crystal structures, 2H-MoS_2_ exhibits thermal stability and semiconductor characteristics, making it the most widely used in practical applications. While 1T-MoS_2_ shows paramagnetism and metallicity [40], it shows metastability and is easy to convert into 2H-MoS_2_, which facilitates the photoelectric application of MoS_2_ [41]. In addition, the number of layers can also alter the electrical and optical properties of MoS_2_ nanosheets. For example, reducing the thickness of MoS_2_ nanosheets can improve conductivity and accelerate the electron transfer rate [42]. The application of MoS_2_ in electronic devices is inseparable from its electronic band structure and electronic properties that are dependent on the density of states. Kuc et al. calculated the electronic band structure of MoS_2_, and obtained the electronic band structure diagram of MoS_2_ with different thicknesses (Figure 1b). The indirect band gap of blocky MoS_2_ is 1.2 eV. During the change from a blocky structure to a layered structure, the band gap thickness gradually increases (1.2–1.9 eV) and transforms from indirect to direct band gap (1.9 eV), showing good absorption and photoluminescence characteristics [43,44]. Compared with graphene with a 0 eV band gap, this adjustable band gap improves the application of MoS_2_ materials in the field of optoelectronics [45]. The key reason for the widespread application of MoS_2_ in optoelectronics is that it exhibits tunable band gap characteristics as it changes in size and structure. Different band gaps bring adjustable optical responsiveness, specific detection rate, and response time, thus having a wide range of applications [46].

**Figure 2:**
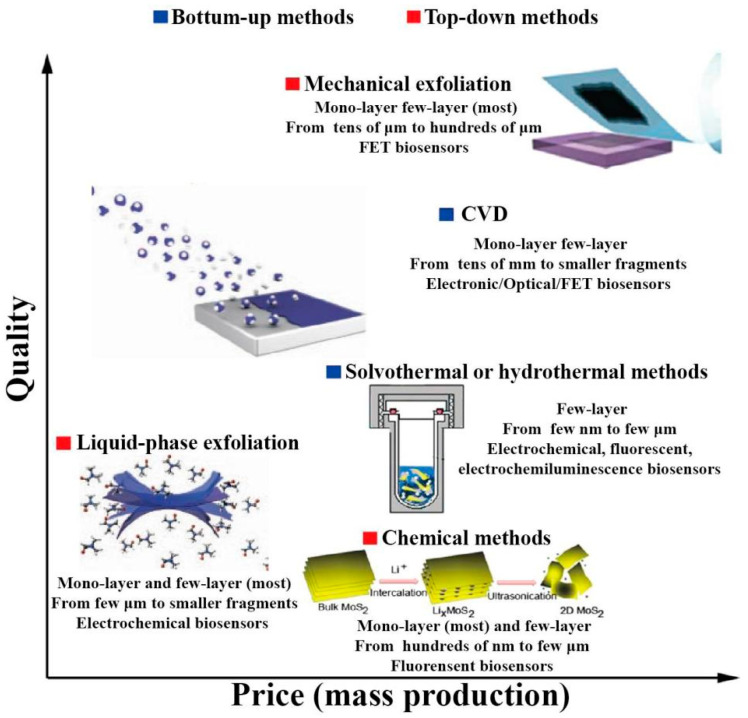
Synthesis methods of MoS_2_ [27,53]. Copyright 2017, Elsevier. Copyright 2012, Springer Nature.

By controlling the synthesis conditions of MoS_2_, various nanostructures can be synthesized. In addition to the most widely used 2D MoS_2_ nanosheets [47], these also include structures such as 0D quantum dots (QD) [48], 1D (nanotubes) [49], and 3D (nanoflowers) [50]. Various structures exhibit different characteristics, such as the direct band gap of nanosheets, photoluminescence of quantum dots, and the high surface area and volume ratio of nanoflowers, which make MoS_2_ exhibit a unique and excellent performance in different application scenarios [51]. The synthesis methods of MoS_2_ can be roughly divided into two categories: top-down and bottom-up (Figure 2). The top-down methods mainly include mechanical exfoliation, liquid-phase exfoliation, and chemical methods. The bottom-up methods mainly include chemical vapor deposition (CVD) and solvothermal or hydrothermal methods [52].

### 2.1. Top-Down Methods

Mechanical exfoliation: As the MoS_2_ layers are connected by a weak van der Waals force, the layered MoS_2_ can be easily peeled off by friction of the bulk substrate MoS_2_ with tape. The peeled tape is pressed into the appropriate substrate, and the synthetic process of MoS_2_ mechanical exfoliation is completed by using the van der Waals force formed between MoS_2_ and the substrate. The mechanical exfoliation is limited by its low yield and thus cannot be used for large-scale production. However, this method can produce layered MoS_2_ with an ideal purity and large transverse size, and is often used as a laboratory preparation method for studying the properties of MoS_2_ materials [54].

Liquid-phase exfoliation: In order to overcome the low yield and difficulty in the large-scale production of mechanical exfoliation, the process of stripping MoS_2_ in solution is called liquid-phase exfoliation. Specifically, the bulk MoS_2_ is dissolved in a suitable solution and separated from the layered MoS_2_ by methods such as bubbling, ultrasonic dispersion, grinding, and shearing [55,56,57,58]. Among them, adding surfactants [59] or bubbling bubbles [60] in the solution can help improve the stability of MoS_2_, effectively maintain the layered structure of MoS_2_ after peeling, and prevent it from recombining with the bulk MoS_2_. Overall, liquid-phase exfoliation is simpler and more cost-effective than mechanical exfoliation, making it stand out in industrial applications [61].

Chemical methods: Chemical methods mainly rely on metal ions entering the interlayer of bulk MoS_2_ to achieve the detachment of MoS_2_ [62]. In the chemical stripping process, the most widely used metal ion reported is Li^+^. However, the intercalation of Li^+^ will simultaneously lead to the transfer of MoS_2_ from the 2H phase exhibiting semiconductor properties, to the 1T phase exhibiting metal properties, which limits the application scenarios of the generated MoS_2_. Further research has shown that annealing at 300 ℃ can restore MoS_2_ formed by Li^+^ intercalation to the 2T phase [63]. Electrochemical methods have also emerged in the MoS_2_ stripping process, typically using block MoS_2_ as the cathode to achieve stripping. However, the large size and area of MoS_2_ produced by electrochemical stripping methods are limited in their application in the biological field, thus requiring more exploration [64].

### 2.2. Bottom-Up Methods

CVD: CVD is a typical nanomaterial growth technique that can be used to prepare high-quality MoS_2_ with scalable size, controllable thickness, and excellent electronic properties [65]. CVD uses precursor gas molecules to adsorb on the substrate surface and generate a thermal chemical decomposition reaction, thus forming high-quality layered MoS_2_. At present, Mo precursors commonly used in CVD mainly include Mo and Molybdenum trioxide, while S precursors mainly use hydrogen sulfide gas or vaporized S [66,67]. On this basis, we further studied the CVD process of MoS_2_ using Molybdenum (V) chloride as a precursor. The characterization results verified that this precursor can produce a larger area of high-quality single-layer MoS_2_ films, but more precursors that can be used for CVD still need to be further explored [68]. CVD can not only produce MoS_2_ with a high film quality, but also easily functionalize MoS_2_ by introducing other precursors during the preparation process. The entire process is very simple and convenient, and is commonly used for studying the properties of MoS_2_ materials and constructing biosensors [69].

Solvothermal or hydrothermal methods: Solvothermal or hydrothermal methods are a simple, scalable, and easily controllable method for preparing MoS_2_. The solvothermal method commonly uses solid precursors, while the hydrothermal method commonly uses liquid precursors [70]. Generally, under high temperature conditions, the molybdate that provides the Mo source and the sulfides that provide the S source are reacted in a polytetrafluoroethylene high-pressure reactor, and MoS_2_ is synthesized through the generated steam pressure. In most cases, an annealing treatment is required to improve its crystal quality and purity [71]. This method can effectively preserve the 2H phase of MoS_2_ and achieve control of the size parameters of MoS_2_ to a certain degree. The harmless preparation process further preserves the biocompatibility of MoS_2_, making it easier to obtain smaller microcrystals with high catalytic activity compared to CVD. It has great potential in constructing electrochemical and fluorescent biosensors [72].

In addition to the above methods, there are also some methods for the preparation of MoS_2_, such as physical vapor deposition (PVD), sputtering, vapor solid growth, etc. Due to the different principles and processes of preparation methods, the generated MoS_2_ exhibits different characteristics and is applied in different scenarios accordingly. The large size of MoS_2_ prepared by mechanical exfoliation limits its biosensing applications. Similarly, PVD exhibits harmful reverse defects compared with CVD during functionalization, which also affects its application range. Correspondingly, CVD and solution chemistry processes have been more widely promoted due to their adjustable preparation process and high-quality synthesis of MoS_2_. The construction of electrochemical/optical sensors using the electrical/optical properties of MoS_2_ has been extensively reported.

## 3. Electrochemical Biosensors for Cancer Biomarkers Detection Based on MoS_2_

Electrochemical sensors are mainly composed of sensitive components, signal transduction components, and nano modified electrode structures. Electrochemical analysis technology is an important detection method in the field of biomedicine. Its basic principle is to analyse the changes in current or impedance signals generated by the interaction between the analyte and the electrode surface. It can monitor the charge movement between reaction interfaces and has significant advantages through its fast response [73]. In recent years, sensitive electrochemical biosensors have been developed for the detection of cancer biomarkers [74]. MoS_2_ has a hexagonal lattice layered structure, which gives it excellent properties such as a high specific surface area, high electron mobility, thermal stability, catalytic activity, and diamagnetism, which is commonly used in semiconductor materials, catalysts, and lubricating materials, etc. [75,76,77]. The unique adjustable bandgap characteristic of MoS_2_ provides excellent photoluminescence properties, which are widely used in optical devices such as photodetectors. Additionally, MoS_2_, as a promising emerging nanomaterial, has low manufacturing costs, rich nanostructures, and is easy to functionalize, making it form hybrid structures with other precious metal nanomaterials, which is widely used in the field of electrochemical sensing [78]. In this chapter, we divide electrochemical biosensors into potentiometry, amperometry, impedimetry, and photoelectrochemical (PEC) biosensors according to different signal transductions, and introduce the latest application progress of MoS_2_ in cancer biomarker detection.

### 3.1. Potentiometry

Potentiometric sensors obtain information about analytes by measuring the current when potential changes, mainly including chronoamperometry (CA), cyclic voltammetry (CV), differential pulse voltammetry (DPV), and square wave voltammetry (SWV), which are widely used electrochemical analysis methods. These methods fix the biometric elements (such as antibodies, enzymes and aptamers) on the electrode surface, and monitor the current changes triggered when the analyte combines with the biometric element when the potential between the working electrode and the reference electrode remains constant. Within the linear potential range, the monitored peak current value is directly related to the concentration of the target analyte in the solution, so as to realize the detection of the target.

**Figure 3:**
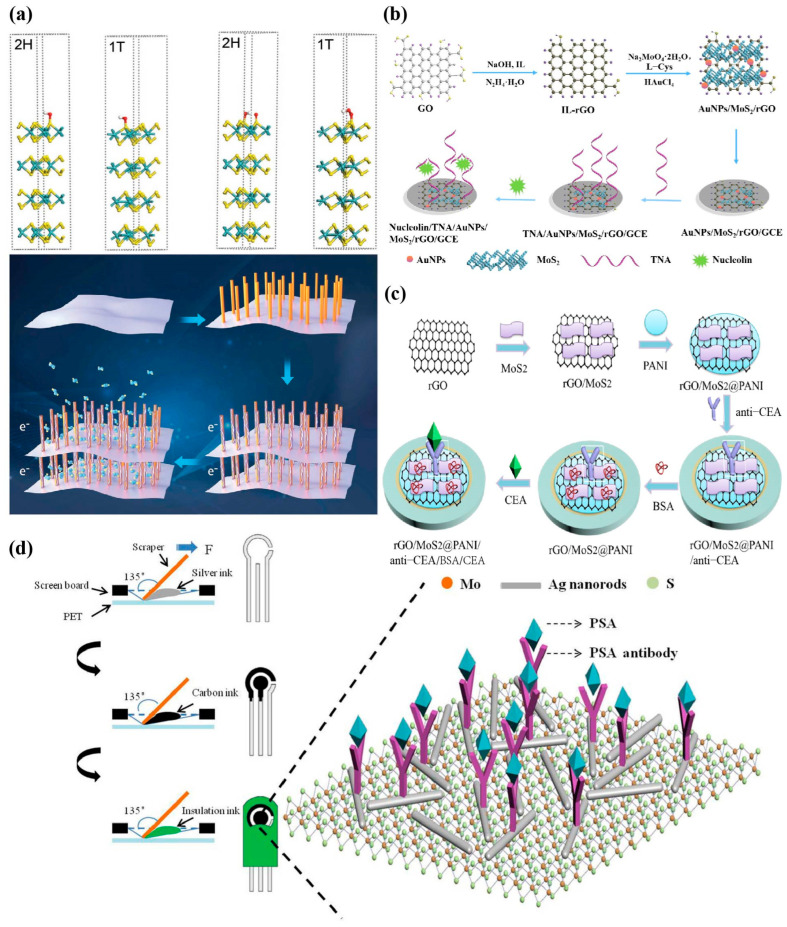
Examples of potentiometric sensors used for detecting various cancer biomarkers. (**a**) Optimized structure of 2H−MoS_2_ and 1T−MoS_2_ (upper plate) and schematic diagram using Pt NWs arrays as nanopillars in ultra-thin MoS_2_ films (lower plate) [80]. Copyright 2020, Wiley. (**b**) Schematic illustration for the synthesis of the AuNPs/MoS2/rGO nanocomposite and nucleolin electrochemical aptasensor strategy [82]. Copyright 2020, Elsevier. (**c**) Schematic diagram of highly sensitive detection of CEA based on rGO/MoS_2_@polyaniline nanosheets of 3D arrays [84]. Copyright 2020, Elsevier. (**d**) Schematic illustration of the fabrication and PSA detection process of EI [85]. Copyright 2020, Springer Nature.

The electronic properties of MoS_2_ are highly dependent on its phase structure. The ultra-thin MoS_2_ has a good performance, but it is difficult to maintain stability in an independent state and is easy to aggregate [79]. In order to improve this problem, Ying et al. [80] used liquid-phase exfoliation and surface modification to synthesize 2H-MoS_2_ (Figure 3a), and used platinum nanowire (Pt NWs) arrays as nanopillars, which were added to the ultra-thin 2D MoS_2_ interlayer to form Pt NWs arrays@MoS_2_ nano hybrid, which improved the specific surface area and porosity, and could be used as “electronic wires” to catalyze electron transfer at the interface, avoiding folding by creating new dimensions. Thus, stability and current signal enhancement were achieved.

Two main reasons that limit the practical application of MoS_2_ in electrochemical sensing are that the strong van der Waals force effect between layers, which leads to aggregation and relatively low conductivity in layers [81]. In order to overcome these shortcomings, Su et al. [82] synthesized ionic liquid (IL) functionalized AuNPs/MoS_2_/rGO nanocomposites for sensitive detection of cancer-specific target nucleolin (Figure 3b). The linear range of the unlabeled electrochemical sensor obtained was 0.5 nM–1.0 μM, and the detection limit was 0.16 nM. Graphene has a large π electronic structure and edge, because of the synergistic effect, and the combination of MoS_2_ and graphene can significantly improve the conductivity and large surface area of MoS_2_ [83]. In addition, the introduction of AuNPs into nanocomposites can not only fix the thioaptamer through the Au-S bond, which improves the affinity and specificity, but it can also enhance electron transfer and amplify the electrochemical signal.

Song et al. [84] modified rGO/MoS_2_@polyaniline nanosheets of 3D arrays on the surface of the Au electrode and further incubated carcinoembryonic antigen (CEA)-specific antibodies to achieve high sensitivity detection of CEA (Figure 3c). First, the sensor combines rGO with MoS_2_ to improve the stack phenomenon of MoS_2_, thus effectively enhancing the electron transfer efficiency of the electrode. Then, polyaniline was further embedded to introduce a large number of amino groups that can bind to CEA specific antibodies, and CEA was detected using CV in a wide linear range (0.001–80 ng/mL), with a limit of detection (LOD) of 0.3 pg/mL. Gui et al. [85] synthesized ce-MoS_2_/AgNR composites by using the van der Waals force and electrostatic interaction between chemically exfoliate MoS_2_ nanosheets (ce-MoS_2_) and Ag nanorods (AgNRs). Because of the synergistic effect, the conductivity of ce-MoS_2_/AgNR composites increased by nearly twice. The prepared unlabeled electrochemical immunosensor (EI) can sensitively detect prostate-specific antigen (PSA) in a wide linear range (0.1–1000 ng/mL) (Figure 3d), with a detection limit as low as 0.051 ng/mL, and could have broad application potential in the clinical diagnosis of prostate cancer.

### 3.2. Amperometry

**Figure 4:**
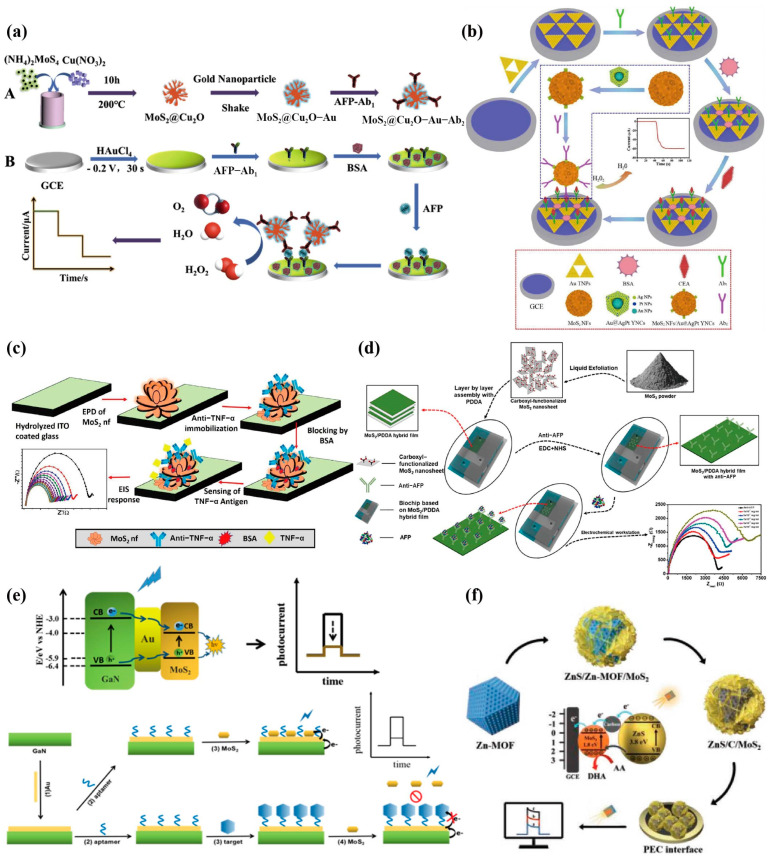
Examples of amperometric/impedance/PEC sensors used for detecting various cancer biomarkers. (**a**) Schematic diagram of ultrasensitive electrochemical immunosensor for AFP detection based on MoS_2_@Cu_2_O−Au [86]. Copyright 2019, Elsevier. (**b**) Schematic illustration of electrochemical immunosensor based on MoS_2_ NFs/Au@AgPt YNCs as the signal amplification label for sensitive detection of CEA [87]. Copyright 2019, Elsevier. (**c**) Schematic representation of BSA/anti−TNF−α/MoS_2_ NFs/ITO immunoelectrode fabrication [89]. Copyright 2022, Elsevier. (**d**) Schematic illustration for self-assembly of MoS_2_/PDDA hybrid film, microfluidic chip fabrication, anti-AFP immobilization, and electrochemical detection of AFP [90]. Copyright 2020, Elsevier. (**e**) Charge-transfer mechanism in MoS_2_/Au/GaN and the AFP detection schematic illustration of the PEC sensor [91]. Copyright 2021, American Chemical Society. (**f**) Schematic illustration of the stepwise fabrication of ZnS/C/MoS_2_ nanocomposite and the enhancement effect on the photocurrent response [92]. Copyright 2019, Wiley.

Amperometric sensors achieve quantitative detection of analytes by applying a constant voltage to the sensing platform to detect the current generated by the conversion of corresponding electroactive substances. Because of their convenience and high accuracy, they are widely used in the detection of cancer biomarkers. As a result of the excellent catalytic activity of MoS_2_ for the reduction in H_2_O_2_, Ma et al. [86] used the hydrothermal method to combine MoS_2_ nanoflowers (MoS_2_ NFs) with p-type metal semiconductor oxide cuprous oxide (MoS_2_@Cu_2_O) (Figure 4a), and, at the same time, the introduction of AuNPs generated MoS_2_@Cu_2_O-Au complexes by Au-S bonds as nanoprobes for signal amplification. The constructed sandwich immunosensor could detect the cancer marker alpha fetoprotein (AFP) of primary liver cancer in the wide linear range of 0.1 pg/mL to 50 ng/mL, demonstrating good application prospects. Ma et al. [87] prepared a sandwich-type electrochemical immunosensor for the sensitive detection of CEA by coupling tri-metallic yolk−shell Au@AgPt nanocubes (Au@AgPt YNCs) loaded on amino-functionalized MoS_2_ NFs (MoS_2_ NFs/Au@AgPt YNCs) with secondary antibodies (Figure 4b). As a result of the biphasic synergistic catalysis, the synthesized MoS_2_ NFs/Au@AgPt YNCs as a signal label effectively catalyzed the reduction of H_2_O_2_ to amplify the current signal, and realized the high-precision detection of CEA in the range of 10 fg/mL–100 ng/mL, with an LOD as low as 3.09 fg/mL (S/N = 3). These works provide ideas for the composite modification of MoS_2_ with different nano forms and further applications in biosensing platforms.

### 3.3. Impedimetry

Impedance sensors are an important type of electrochemical sensing that obtains information about analytes by measuring the conductance through interface reactions on the electrode surface. This type of sensor is very sensitive to the change in electrode, and is in an advantageous position in the detection of biomarkers. Therefore, it is also widely introduced into the construction of the electrochemical sensing platform for the detection of cancer biomarkers. Jia et al. [88] prepared a novel nanohybrid of polyoxometalate-derived MoS_2_ nanosheets (pd-MoS_2_ NSs) using a hydrothermal method, which exhibited an excellent electrochemical activity and abundant catalytic sites. Furthermore, pd-MoS_2_ NSs were vertically grown over β-FeOOH NRs (pd-MoS_2_@β-FeOOH), serving as complementary DNA platforms for fixing oncogenes and tumour suppressor miRNA-21, using electrochemical impedance spectroscopy (EIS) to detect miRNA-21, with an LOD as low as 0.11 fM. In addition, microfluidic electrochemical immunochips have been evaluated as a powerful detection platform because of their high sensitivity, low cost, portability, and easy miniaturization. Sri et al. [89] synthesized MoS_2_ NFs using the same method, and electrophoretically deposited them on an indium tin oxide (ITO)-coated glass substrate. Because of the morphology of MoS_2_ NFs, antibodies can be effectively fixed on the electrode surface through physical adsorption. The biosensor can sensitively detect tumour necrosis factor-α (TNF-α) between 1–200 pg/mL, with an LOD as low as 0.202 pg/mL (Figure 4c). Hu et al. [90] first prepared MoS_2_ by liquid-phase exfoliation and formed a hybrid film with PDDA, designed a three-electrode system in the microfluidic chip, and introduced a MoS_2_/PDDA film modified with anti-AFP as the working electrode, Ag/AgCl as the reference electrode, and ITO as the counter electrode (Figure 4d). The linear range of AFP detected by EIS was 0.1 ng/mL to 10 ng/mL, with an LOD of 0.033 ng/mL.

### 3.4. Photoelectrochemistry (PEC)

PEC utilizes photosensitive materials at the electrode interface as signal converters to analyse the electrical signals generated by analytes under light irradiation, combining the advantages of spectral analysis and electrochemical technology. MoS_2_ exhibits excellent characteristics of a tunable bandgap in its transition from a blocky structure to a layered structure. The quantum confinement effect led to good visible light absorption and photoelectric conversion efficiency of layered MoS_2_ as a direct bandgap semiconductor under visible light excitation, resulting in photocurrent generation. Therefore, it has been introduced into the application of photoelectrochemical sensing platforms. Hu et al. [91] utilized this mechanism to design a PEC sensing platform based on MoS_2_/Au/GaN for the high sensitivity detection of AFP (Figure 4e). MoS_2_ can suppress the charge transfer of Au/GaN photoelectrodes, leading to a significant decrease in photocurrent. However, the presence of AFP can reduce the inhibitory effect on the photocurrent, thereby utilizing the difference in photocurrent to detect AFP. AFP detection is achieved in a wide linear range of 1.0–150 ng/mL, with an LOD of 0.3 ng/mL. This method has a good sensitivity and high selectivity for AFP detection. Wei et al. [92] synthesized a light-responsive ZnS/C/MoS_2_ nanocomposite to construct a PEC immunosensor for detecting CEA, with a linear range of 2.0 pg/mL–10.0 ng/mL and an LOD of 1.30 pg/mL (S/N = 3), showing good analytical characteristics (Figure 4f). In addition to the above sensing methods, other sensing methods based on MoS_2_ are listed in Table 1 to detect various cancer biomarkers.

## 4. Optical Biosensors for Cancer Biomarker Detection Based on MoS_2_

Optical biosensors bring additional advantages in the fields of biotechnology, environmental research, disease diagnosis, and medical applications due to their high selectivity, and fast and sensitive measurement. The working principle and key performance indicators of optical biosensors largely depend on optical transducers tightly integrated with biological sensing components [107]. Based on different biosensor elements, optical biosensors are divided into colorimetry, electrochemiluminescence (ECL), fluorescence, surface enhanced Raman scattering (SERS), surface plasmon resonance (SPR), and other sensing methods. In combination with MoS_2_, the latest application progress in cancer biomarker detection is introduced.

### 4.1. Colorimetry

**Figure 5:**
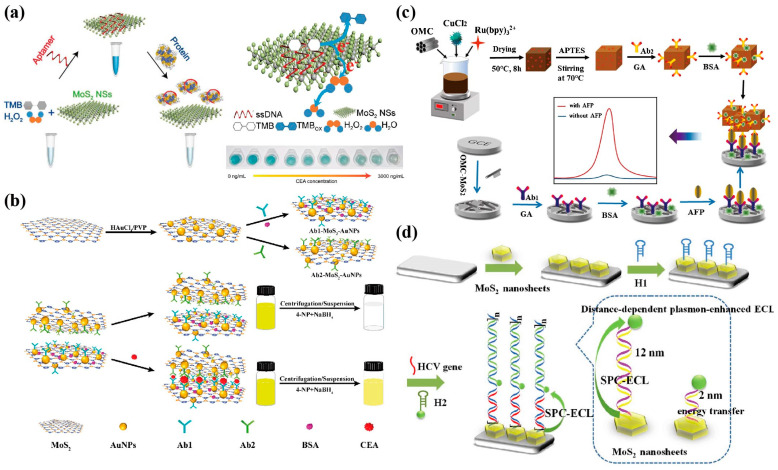
Examples of colorimetric/ECL sensors used for detecting various cancer biomarkers. (**a**) Schematic illustration of the colorimetric biosensor based on aptamer-modified MoS_2_ NSs for CEA protein detection [110]. Copyright 2020, RSC Pub. (**b**) Preparation of MoS_2_-AuNPs nanohybrids and Ab-MoS_2_-AuNPs nanoprobes, and schematic illustration of the colorimetric sensor for CEA detection [111]. Copyright 2019, American Chemical Society. (**c**) Schematic illustration of the sandwich-configuration ECL immunoassay based on Cu_2_O@OMC-Ru (bpy)_3_^2+^ and OMC-MoS_2_ for the determination of AFP [118]. Copyright 2020, Springer Nature. (**d**) The HCR-based sensing process and distance-dependent plasmon-enhanced ECL for HCV detection [120]. Copyright 2020, Elsevier.

MoS_2_, with its large surface area and exposed reaction sites, can be used as a nano enzyme to show the catalytic activity and excellent stability of peroxidase, and it simulates natural enzymes to make the substrate colour change. This feature is used to build a colorimetric sensor to detect cancer biomarkers [108,109]. Zhao et al. [110] introduced an aptamer to enhance the catalytic activity of MoS_2_ NSs on peroxidase substrates and designed a colorimetric sensor for the intuitive detection of CEA, achieving sensitivity detection of CEA by successfully recording absorbance (Figure 5a). The sensor exhibited a linear response in the range of 50 to 1000 ng/mL, with an LOD of 50 ng/mL, demonstrating good specificity and practical application capabilities. Shao et al. [111] utilized the high catalytic activity of MoS_2_-AuNPs nanohybrids to reduce NaBH_4_ to 4-NP and make the yellow solution colourless, and constructed a colorimetric immunosensor for CEA detection (Figure 5b). The absorbance peak intensity of the colorimetric sensor maintained a good linear relationship in the range of 5 pg/mL to 10 ng/mL, with an LOD as low as 0.5 pg/mL. Wang et al. [112] developed a new colorimetric nano biological platform for the efficient and highly sensitive capture of circulating tumour cells (CTC), in which the MoS_2_ NSs surface was modified with two kinds of aptamer functionalized PH sensitive heterochromatic dyes used as a visual detection chip, which had a good PH sensitivity and high dyeing ability.

### 4.2. Electrochemiluminescence (ECL)

ECL is a special form of chemiluminescence caused by the redox between electrogenerated high-energy radicals [113]. It does not rely on external light excitation and avoids the adverse effects of self-luminous and light scattering [114]. Therefore, it has the characteristics of a precise response, easy control, low noise background signal, high sensitivity, good repeatability, and wide linear range, and has become a powerful tool for biomarker detection and clinical diagnosis in recent years [115,116]. MoS_2_ can effectively improve the rate of electron transfer, and it is emerging in the construction of ECL sensing platforms for cancer biomarkers [117]. Zhang et al. [118] used ordered mesoporous carbon-MoS_2_ (OMC-MoS_2_) as a sensing platform and Cu_2_O@OMC-Ru (bpy)_3_^2+^ as signal tags to develop a sandwich ECL immunosensor for the detection of AFP (Figure 5c). As we know, MoS_2_ NSs are easy to agglomerate, resulting in a loss of activity, and the synergistic effect of nanocomposites can offset this loss of activity. OMC exhibits an excellent electrocatalytic performance due to its ordered pore structure, high specific surface area, and high porosity [119]. Therefore, OMC-MoS_2_ can synergistically increase the effective surface area and conductivity to improve sensor sensitivity. The ECL detection range of AFP is 0.1 pg/mL–10 ng/mL, with an LOD of 0.011 pg/mL (S/N = 3). Liu et al. [120] synthesized MoS_2_ NSs using the hydrothermal method, enhanced the electrochemiluminescence signal of sulphur doped boron nitrogen quantum dots (QDs) using its strong surface plasmon coupling (SPC) light absorption effect in visible and near-infrared regions, and constructed an ECL sensing platform amplified by the hybrid chain reaction (HCR) for hepatitis C virus (HCV) genetic testing (Figure 5d).

### 4.3. Fluorescence

**Figure 6:**
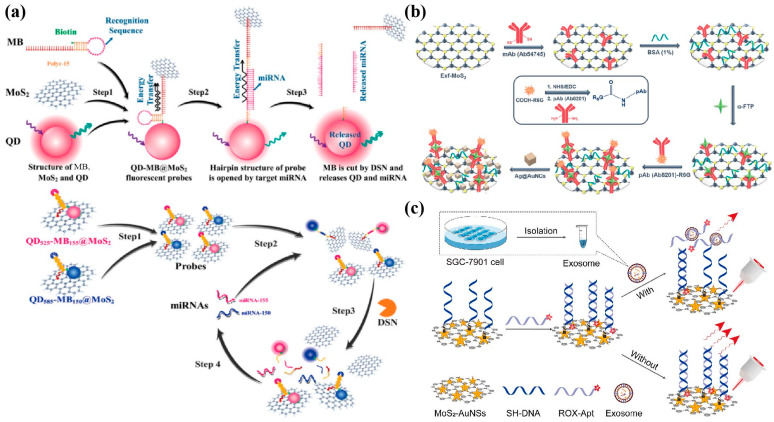
Examples of fluorescence/SERS sensors used for detecting various cancer biomarkers. (**a**) Schematic illustration of dual miRNA detection by QD-MB@MoS_2_ fluorescent probes [124]. Copyright 2022, Elsevier. (**b**) Schematic illustration of the SERS immunosensor based on Au@AgNCs/MoS_2_ nanocomposites [128]. Copyright 2021, American Chemical Society. (**c**) Fabrication of MoS_2_-based aptasensor for exosomes detection [129]. Copyright 2022, Elsevier.

Fluorescence analysis is an advanced analytical method with a high sensitivity, selectivity, and practicality, which can qualitatively and quantitatively analyse the changes in fluorescence intensity, emission spectrum, and fluorescence molecular lifetime of substances [121]. Due to the strong adsorption capacity and wide absorption spectrum of MoS_2_ NSs to ssDNA, MoS_2_ NSs can quench fluorescent groups with different emission wavelengths, showing unique advantages in the construction of fluorescent biosensor platforms [122]. Liang et al. [123] built a fluorescence sensing platform for the hepatocellular carcinoma (HCC) biomarker GP73 based on the Förster resonance energy transfer (FRET). Among them, utilizing the synergistic effect of the MoS_2_@rGO composites as a fluorescence receptor further enhanced the quenching effect, and the nitrogen-doped graphene QDs modified by the GP73 aptamer were used as fluorescence donors. The detection range was 5 ng/mL to 100 ng/mL, and the LOD was 4.54 ng/mL (S/N = 3). It also showed a good detection effect in human serum. Wang et al. [124] designed QD molecular beacons (QD-MBs) functionalized with a MoS_2_ fluorescent probe (QD-MB@MoS_2_) for the dual detection of two kinds of miRNAs related to multiple myeloma (MM), with an LOD as low as the fM level, realizing ultra-high sensitivity detection (Figure 6a). In addition, when the MoS_2_ crystal becomes very thin, the transition from the indirect bandgap to the direct bandgap will produce a strong fluorescence [125]. MoS_2_ QDs have strong quantum confinement and edge effects and other photoelectric properties, and are widely used in fluorescence sensing, catalysis, biological imaging, and other fields [126]. Ge et al., based on the quenching of MoS_2_ QDs by the inner filter effect (IFE) and rolling circle amplification (RCA) technology, constructed a label-free and highly sensitive miRNA fluorescence detection platform with a high selectivity and satisfactory recovery [126].

### 4.4. Surface Enhanced Raman Scattering (SERS)

SERS technology can provide molecular fingerprint information, has a high sensitivity and specificity, and does not cause damage to the sample, and is thus considered as a promising analytical technology in the field of disease analysis [127]. SERS sensor composition mainly include substrates, target detection substances, and SERS capture probes. MoS_2_ has been applied in the preparation of SERS capture probes due to its large specific surface area, stability, and excellent catalytic performance. Engine et al. [128] developed a SERS sandwich immunosensor for the ultra-sensitive detection of AFP (Figure 6b). Among them, MoS_2_ is modified by the monoclonal antibody as the capture probe of AFP, and its high surface area and adsorption capacity for biomolecules make the sensing interface more stable. The SERS immunosensor based on Au@AgNCs/MoS_2_ nanocomposites has a good linear response in the range of 1 pg/mL to 10 ng/mL, with an LOD as low as 0.03 pg/mL. Pan et al. [129] developed a sensitive and direct SERS aptasensor for detecting gastric cancer exosomes. AuNSs-decorated MoS_2_ NSs (MoS_2_-AuNSs) surfaces were assembled with ROX-labelled aptamers (ROX-Apt) used as nano probes to achieve the ultra-sensitive capture of exosomes (Figure 6c). This sensor quantitatively detected gastric cancer exosomes over a wide range of SERS signals (55–5.5 × 10^5^ particles/μL), with an LOD as low as 17 particles/μL, which provides a prospective platform for the early diagnosis of gastric cancer. In addition, Hilal et al. [130] developed a sandwich-type SERS immunosensor for the sensitive detection of CEA, which has a good selectivity and stability and is well applied in clinic.

### 4.5. Surface Plasmon Resonance (SPR)

SPR provides a non-invasive and label-free method to detect analytes. MoS_2_ has a large absorption coefficient and high refractive index at 500 nm, whose structure is conducive to the propagation of the surface plasma. Such photoelectric characteristics enhance SPR signals and improve the sensitivity of the sensor [131]. Therefore, the modification of MoS_2_ is also applied in SPR sensors for cancer biomarker detection. Chiu et al. [132] prepared MoS_2_ by the liquid-phase exfoliation and covalently functionalized it to form carboxyl-functionalized MoS_2_ (carboxyl-MoS_2_) acting as a signal amplification sensing modification layer. The carboxylation modification effectively improved the sensitivity of the SPR sensor. The SPR chip based on carboxyl-MoS_2_ was used to specifically detect the lung cancer-associated biomarker cytokeratin 19 fragment (CYFRA21-1),which shows a wide linear range (0.05 pg/mL–100 ng/mL) and low LOD (0.05 pg/mL), and has a good specificity, selectivity, sensitivity, and affinity. Compared with traditional SPR bare gold chips, the SPR chip has many characteristics, such as a unique glycan matrix structure, high surface area carboxylic acid groups, and excellent biological affinity. In addition to the above sensing methods, other sensing methods based on MoS_2_ are listed in the Table 2 to detect various cancer biomarkers.

## 5. Miscellaneous Biosensors for Cancer Biomarkers Detection Based on MoS_2_

**Figure 7:**
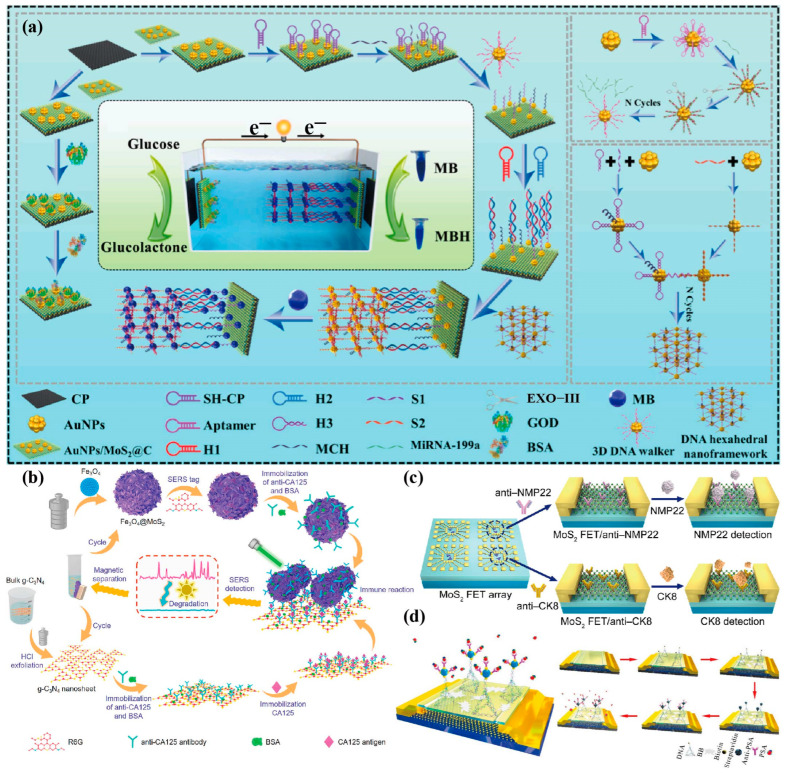
(**a**) Creation of an ultra-sensitive dual-mode approach based on self-powered sensor detecting liver cancer makers [142]. Copyright 2023, Elsevier. (**b**) Fabrication of Fe_3_O_4_@MoS_2_ composites and g-C3N4 NSs as well as the protocol of recyclable SERS-based sandwich immunoassay [143]. Copyright 2023, Elsevier. (**c**) Schematic illustration of MoS_2_ NSs-based FET sensor array for the simultaneous detection of NMP22 and CK8 [144]. Copyright 2020, Springer Nature. (**d**) Surface functionalization and electrical characterization of the MoS_2_ FET device. Schematic diagram showing the 3D structure of the functionalized MoS_2_ biosensor (left plate). Flow chart of the device functionalization process (right plate) [145]. Copyright 2021, Elsevier.

In addition to the two main types of cancer biomarker sensing platforms based on optics and electrochemistry, this section covers some other types of cancer biomarker sensors based on MoS_2_. MoS_2_ has the characteristics of a direct bandgap, excellent switching ratio, and high carrier mobility [141], which makes the MoS_2_ field effect transistor (FET) biosensor have the advantages of a high sensitivity, label-free biological detection, system integration, and easy manufacturing. Recently, Shi et al. [142] used 2D carbon-coated MoS_2_ NRs to form an overlapping hybrid structure (MoS_2_@C), combined with HCR, 3D DNA walker, and DNA hexahedron nano framework, which established a novel four-fold amplification and self-powered intelligent sensing platform for the ultra-sensitive colorimetric/electrochemical dual-mode detection of tumour suppressor miRNAs in HCC (Figure 7a). As a hollow network structure, MoS_2_@C can extend the interlayer distance, not only improving the stability of the materials, but also providing more enzymes and probe binding sites. This sensor quantifies the concentration of miRNAs by measuring the open circuit voltage and colour changes of MBs, making it an efficient, sensitive, and highly specific sensing method. Wang et al. [143] mixed metals and semiconductors to make MoS_2_ composite (1T-MoS_2_) as an excellent substrate with a high conductivity and electronic density of state, and combined it with the 2D graphitic carbon nitride (g-C_3_N_4_) to build a recyclable immunoassay platform for CA125 (Figure 7b), with an LOD as low as 4.96 × 10^−4^ IU/mL. Based on the excellent electronic properties and high specific recognition ability of MoS_2_ NSs, Yang et al. [144] constructed a FET sensor array to detect the bladder cancer biomarkers nuclear matrix protein 22 (NMP22) and cytokeratin 8 (CK8), and achieved ultra-sensitive detection in a wide linear range (10^−6^–10^−1^ pg/mL), with an LOD as low as 0.027 aM and 0.019 aM (Figure 7c). Zhang et al. [145] functionalized MoS_2_ FET by coupling DNA tetrahedron and biotin-streptavidin, and detected PSA in the range of 1 fg/mL–100 ng/mL, with broad development prospects in the field of real-time detection (Figure 7d).

**Figure 8:**
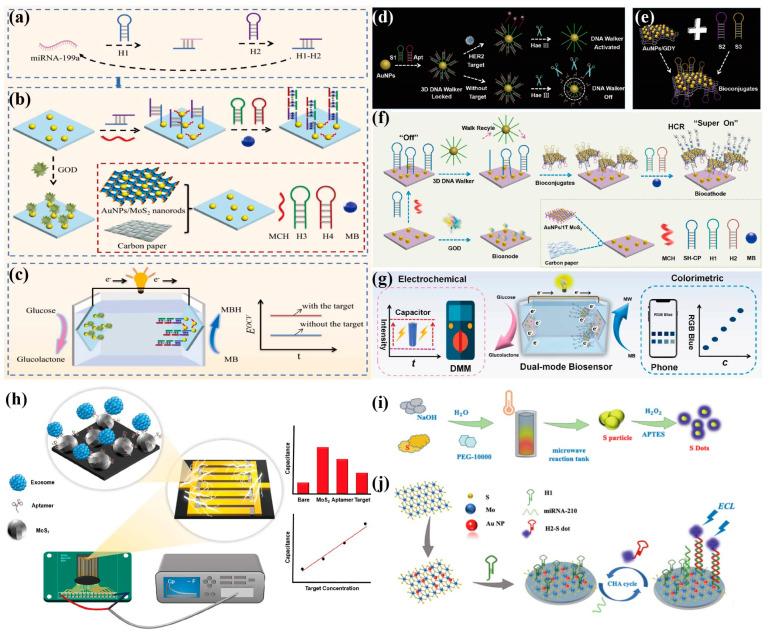
Detail diagram of the preparation process of the self-powered biomolecular sensing system [147]. (**a**) The CHA process involving the target object. (**b**) The establishment process of the bioanode and bio-cathode. (**c**) Schematic representation of the assembly of the self-powered biosensor. Copyright 2023, Elsevier. Schematic illustration of dual-mode self-powered biosensing platform [148]. (**d**) HER2 recognition activates 3D DNA walker. (**e**) Preparation of bioconjugates. (**f**) Assembly process of biological electrodes. (**g**) The detection mechanism of the dual-mode strategy. Copyright 2023, Elsevier. (**h**) Schematic image of an exosome detection biosensor [149]. Copyright 2022, Elsevier. Illustration of (**i**) the synthesis of S dots and (**j**) the ECL sensing system based on S dots and Au NP@MoS_2_ nanosheet heterostructure [136]. Copyright 2022, Elsevier.

In recent years, aptasensors have become a major class of sensor technology. Aptamers are single stranded nucleotides (ssDNA or ssRNA) synthesized through systematic evolution of ligands by exponential enrichment (SELEX). With different sequences and 3D structures, they can specifically bind to various targets, such as small molecules, proteins, whole cells, and nucleotide sequences [146]. Aptamers are “chemical antibodies” that detect analytes based on conformational changes, which have the advantages of a small size, high chemical stability, high specificity, easy synthesis and modification, and low cost, etc., and which are widely used in the field of novel biosensing technologies in combination with numerous nanomaterials. Shi et al. [147] constructed a biofuel cell based on a hybridization chain reaction and catalytic hairpin assembly (CHA) self-assembly, and developed an electrochemical/colorimetric dual mode biosensing platform for identifying the colon cancer inhibitory factor miRNA-199a (Figure 8a–c). The bioanode of this biofuel cell is a flexible carbon cloth loaded with glucose oxidized-functionalized MoS_2_ NRs, and the bio-cathode is composed of double stranded deoxyribonucleotide chains generated by nucleic acid amplification technology. The combination of high surface area MoS_2_ NRs, enzyme-free cascade signal amplification technology, and non-interference dual mode detection strategy makes the sensor highly sensitive, selective, and accurate. The sensor has an electrochemical linear range of 0.1 fM–100 pM, LOD of 24.1 aM (S/N = 3), colorimetric linear range of 0.1 fM–10,000 pM, and LOD of 34.5 aM (S/N = 3), is a promising method for detecting colorectal cancer inhibitory factors. Similarly, Hou et al. [148] also developed a self-powered biosensor based on ultrasensitive enzyme biofuel cell in the same year for electrochemical/colorimetric dual mode detection of HER2 (Figure 8d–g). The selected construction materials for this biofuel cell were 1T-MoS_2_ and graphdiyne, among which 1T-MoS_2_ is a metal phase of MoS_2_, which has an extraordinary conductivity, enlarged interlayer spacing, and abundant reaction sites, greatly improving the performance of the battery. Furthermore, Lee et al. [149] developed a DNA aptamer/MoS_2_ heterolayer electrobiosensor and achieved ultra-sensitive early diagnosis of exosomes combined with an interdigitated microgap electrode (IDMGE) system (Figure 8h). MoS_2_ nanoparticles have a good biomolecular detection ability, more effectively immobilizing the aptamer and improving the electrical sensitivity of the sensor. Based on the work function tuning strategy, Hou et al. [136] constructed an AuNPs@MoS_2_ NSs heterostructure with a large specific surface area, good biocompatibility, good electrocatalytic activity, and high conductivity, and developed a new ECL sensor for the detection of miRNA-210 in triple negative breast cancer tissues (Figure 8i,j). Due to the small bandgap of MoS_2_ NSs, the ECL quenching ability of AuNPs by close range electron transfer can be effectively suppressed in the heterostructure, while also improving the conductivity and LSPR performance of AuNPs. In addition to the above sensing methods, other sensing methods for detecting various cancer biomarkers based on MoS_2_ are listed in Table 3.

## 6. Discussion and Outlook

As a representative of TMDs, the unique bandgap adjustable layered structure of MoS_2_ has shown excellent optical, electronic, and mechanical properties in the construction of sensing interfaces, and has further expanded the application of MoS_2_ as a sensing electrode in different functionalization processes. It is a promising nanomaterial to replace GO and other semiconductor devices. According to the above, the composite-based sensing interfaces built on MoS_2_ have been widely used in the detection of different cancer biomarkers. These sensing platforms have shown a good sensitivity, specificity, and reproducibility, comprehensively proving the great potential of MoS_2_ in early cancer screening. However, MoS_2_ still faces severe challenges and further development.

As the growth process of MoS_2_ is uncontrollable, how to reduce the influence of impurities and lattice defects and find a large-scale and high-quality synthesis method are the fundamental issues of MoS_2_. Two-dimensional MoS_2_ has the defect of easy aggregation, which leads to a reduction in electrochemical activity. Accurate control of the synthesis of MoS_2_ with a uniform thickness, ideal size, and colloidal stability still needs further exploration. Although these shortages can be improved by modifying MoS_2_ with molecules, nanoparticles, or single-atom sites to form composites, achieving controllable parameters such as shape, size, charge, stability, and surface chemistry during the design process remains a major challenge [163]. Therefore, advanced synthesis techniques are needed to address these issues, such as exploring surface constrained synthesis methods and exploring better exfoliative conditions using material informatics prediction methods [164]. The covalent/non-covalent functionalization of MoS_2_ is a powerful tool to improve the dispersion and stability, and to effectively expand its application by adjusting the physical properties or adding new attributes [78]. For example, changing the MoS_2_ ratio of 2H and 1T crystal structures by inducing defects or introducing additional negative charges, as well as changing the MoS_2_ bandgap through doping, embedding, and other methods [41]. By using these approaches, MoS_2_ can be promoted to achieve low-cost, reliable, and large-scale production, which will effectively expand the construction and application capabilities of composite-based sensing interfaces based on MoS_2_. These new developments will drive different composite-based sensing interfaces based on MoS_2_ into a new era.

The conversion of MoS_2_ between different dimensions makes its characteristics and applications rich, for example as semiconductor, metal, or superconducting materials. For example, 2H-MoS_2_ and 3R-MoS_2_ can be used for dry lubricating oil [75]. The nonlinear optical properties of 3R-MoS_2_ can be used for quantum measurements and nonlinear optical quality sensing in the biomedical field. Although MoS_2_ shows good electronic properties, compared with silicon, its electron mobility is lower and its bandgap is higher, which still creates many problems in the construction of MoS_2_ FET sensors. The conductivity of MoS_2_ NSs is affected by temperature and thickness, which increases with increasing temperature and decreases with increasing thickness until it reaches a 3D structure [76]. Furthermore, MoS_2_ also has good characteristics of electron-spin and magnetoresistance. The research shows that MoS_2_ has half-metallic ferromagnetism when doped with Sc, and has a unified spin-polarization value, which is conducive to the development of spintronics [77]. The key factor for the wide application of MoS_2_ in optoelectronics is that it has an adjustable bandgap that changes with size and structure. The change in the bandgap dimension leads to a change in photoluminescence characteristics. MoS_2_ QDs have a higher bandgap than MoS_2_ NSs, whose optical properties can be changed by adjusting the size, or the photoluminescence intensity, and the emission rate can be enhanced according to the light−matter interaction. MoS_2_ can realize broadband detection from visible light to far infrared, which is of great significance for safety, biosensors, and thermal imaging, but its responsivity and detection rate are poor due to poor light absorption and large dark current [165]. We also found that the quenching ability of MoS_2_ is easily affected by water and oxygen in the medium. Additionally, although MoS_2_ has good biocompatibility, bioabsorption, anti-cancer, and antibacterial effects in catalytic and biological activity applications, it also has a high toxicity [166].

In this work, the applications of electrochemical and optical sensing platforms based on MoS_2_ in the field of early cancer diagnosis are reviewed. In fact, MoS_2_ shines brightly in many fields due to its rich and colourful excellent characteristics. As an example, MoS_2_ is a suitable battery electrode material, can be used for hydrogen evolution reactions, has great application prospects in biomedical fields such as cancer treatment and relevant fields of the Internet of Things, and even performs a certain function in emerging technological fields such as microwave and terahertz technology. Furthermore, the miniaturization of the sensing technology is of great significance for point of care testing (POCT). Now, with the popularity of miniaturized electronic devices, wearable, portable devices, electronic skin, and other emerging technologies have been developed rapidly. MoS_2_, with its good mechanical properties, has become the best candidate material for flexible sensors that can be attached to the skin to achieve non-invasive detection of biomarkers in body fluids, thus minimizing skin irritation and making it easy to measure [167]. The detection of cancer biomarkers in sweat and other bodily fluids (such as saliva and urine) based on MoS_2_ flexible sensing interfaces needs further development, which cannot be separated from the development of electronic circuits, wireless communication units, and power supply systems.

## 7. Conclusions

In this review, we summarize the application of MoS_2_-based electrochemical and optical sensing platforms in the field of early cancer diagnosis, and explore the improvement and application of the MoS_2_ synthesis process and material properties in the construction of biosensor platforms, and focus on the excellent characteristics of MoS_2_ and its composite materials in the construction of electrochemical and optical sensing platforms, as well as their applications in the field of cancer biomarkers detection. Nowadays, although MoS_2_ still faces significant challenges in synthesis technology, enhancement of dispersion, and conductivity, the interdisciplinary development of materials informatics and other disciplines has led to an increasing number of material synthesis methods being used for the study of MoS_2_. It has been proven that MoS_2_ plays a crucial role in healthcare, optoelectronics, energy, chemical, and mechanical industries.

## Figures and Tables

**Table 1:** Electrochemical biosensors for cancer biomarkers detection based on MoS_2_.

Method	Analytes	Electrode/Label	Linear Range	LOD	Ref.
Chronoamperometry	AMACR	Pt NWs array@2H-MoS_2_/SPE	0.70–12.50 ng/µL	0.5 pg/µL	[80]
CV	CEA	Ab/rGO/MoS_2_@PANI/GCE	0.001–80 ng/mL	0.3 pg/mL	[84]
CV	PSA	Ab/ce-MoS_2_/AgNR/SPE	0.1–1000 ng/mL	0.051 ng/mL	[85]
DPV	CEA	Ab/MoS_2_-PBNCs/GCE	0.005–10 ng/mL	0.54 pg/mL	[93]
DPV	CEA	Ag/MoS_2_@Fe_3_O_4_-Ab2/CEA/Ab1/Ag/MGCE	0.0001–20 ng/mL	0.03 pg/mL	[94]
DPV	miRNA-182	ssRNA/MoS_2_/Ti_3_C_2_/GCE	1 fM–0.1 nM	0.43 fM	[95]
DPV	CEA	MoS_2_-AuNPs/HRP-Ab2/CEA/Ab1/MoS_2_-AuNPs/GCE	10 fg/mL–1 ng/mL	1.2 fg/mL	[96]
DPV	Nucleolin	TNA/AuNPs/MoS_2_/rGO/GCE	0.5 nM–1.0 μM	0.16 nM	[82]
DPV	CEA	Ab/Pd@Pt/MoS_2_-Gr/GCE	0.00001–100 ng/mL	0.005 pg/mL	[97]
DPV	CA125	Ab/CuBTC@MoS_2_-AuNPs/SPE	0.5 mU/mL–500 U/mL	0.5 mU/mL	[98]
DPV	anti-retroviral agent indinavir	ZnO NRs/MoS_2_ NSs/SPE	0.01–0.66 μM & 0.66–7.88 μM	0.007 μM	[99]
SWV	CEA	CeO2-MoS_2_-Pb^2+^-Ab2/CEA/Ab1/AuNPs/GCE	0.001–80 ng/mL	0.3 pg/mL	[100]
Amperometry	CEA	Ab/Ag/MoS_2_/rGO/GCE	0.01 pg/mL–100 ng/mL	1.6 fg/mL	[101]
Amperometry	HBeAg	Au@Pd/MoS_2_@MWCNTs-Ab2/HBeAg/Ab1/p-GO@Au/GCE	0.1–500 pg/mL	26 fg/mL	[102]
Amperometry	AFP	MoS_2_@Cu_2_O-Au-Ab2/AFP/Ab1/AuNPs/GCE	0.1 pg/mL–50 ng/mL	0.037 pg/mL	[86]
Amperometry	AFP	Ab/Pt NDs/PDDA/MoS_2_@PPy NTs/GCE	50 fg/mL–50 ng/mL	17 fg/mL	[103]
Amperometry	CEA	MoS_2_ NFs/Au@AgPt YNCs -Ab2/CEA/Ab1/AuTNPs/GCE	10 fg/mL–100 ng/mL	3.09 fg/mL	[87]
EIS	CML	pDNA/PANI-MoS_2_/ITO	10^−17^–10^−6^ M	3 × 10^−18^ M	[104]
EIS	AFP	Ab/MoS_2_/PDDA/Ag/AgCl wire	0.1–10 ng/mL	0.033 ng/mL	[90]
EIS	miRNA-21	cDNA/pd-MoS_2_@β-FeOOH/Au	1 fM–5 nM	0.11 fM	[88]
EIS	TNF-α	MoS_2_ NFs	0.01–200 pg/ml	0.202 pg/ml	[89]
PEC	CEA	ALP-Au-Ab2/CEA /Ab1/ZnS/C/MoS_2_/GCE	2.0 pg/mL–10.0 ng/mL	1.30 pg/mL	[92]
PEC	SCCA	Ab/AuNPs/C/MoS_2_/GCE	0.005–8 ng/mL	1.8 pg/mL	[105]
PEC	AFP	DNA/Au/GaN	1.0–150 ng/mL	0.3 ng/mL	[91]
PEC	MCF-7 cells	PM6:Y6/anti-EpCAM-MNs /Au NPs/Au-aptamer	10–10,000 cell/mL	9 cell/mL	[106]

**Table 2:** Optical biosensors for cancer biomarker detection based on MoS_2_.

Method	Analytes	Electrode/Label	Linear Range	LOD	Ref.
Colorimetry	CEA	Au NPs-MoS_2_-Ab2/ CEA/Ab1/MoS_2_-Au NPs	5 pg/mL–10 ng/mL	0.5 pg/mL	[111]
Colorimetry	CEA	DNA/MoS_2_ NSs	50–1000 ng/mL	50 ng/mL	[110]
Colorimetry	CTC	TP/SYL_3_C-MoS_2_	5–10^4^ cells/mL	2 cells/mL	[112]
ECL	CEA	Ab/MOF-545-Zn@MQDs/GCE	0.18–1000 ng/mL	0.45 pg/mL	[133]
ECL	PSA	GOD-SiO_2_-Ab2/PSA /Ab1/MoS_2_-AuNPs/GCE	0.5 pg/mL–10.0 ng/mL	0.20 pg/mL	[134]
ECL	HCV gene	S-BN QDs-hairpin DNA2 (H2)/MoS_2_ Ns	0.5 pmoL/L–1 nmoL/L	0.17 pmoL/L.	[121]
ECL	HPV 16 DNA	Zn-doped MoS_2_ QDs & QD-DNA/reductive Cu(I) particles	0.1–200 nmolL	0.03 nmol/L	[135]
ECL	miRNA-210	S dots/Au NP@MoS_2_ NSs	0.1 pM–10 nM	0.03 pM	[136]
ECL	miRNA-21	luminophore/MoS_2_ QDs@Zeolitic Imidazolate 2 Framework-8	Buffer (0.1 mM PBS), co-reactant (2 mM H_2_O)	14.6 aM	[137]
Fluorescence	CA15-3	DNA/MoS_2_ NSs	0.01–0.1 U/mL	0.0039 U/mL	[138]
Fluorescence	PD-1	MoS_2_-NFP	125–8000 pg/mL	85.5 pg/mL	[139]
Fluorescence	miRNA-155 & miRNA-150	QD-MB @MoS_2_	10 fM–1 nM	7.19 fM & 5.84 fM	[124]
SERS	CEA	MoS_2_ NFs@Au NPs/MBA-Ab2/CEA /Ab1/Fe_3_O_4_ NPs@Au NPs/ d-Ti_3_C_2_TX Mxene	0.0001–100.0 ng/mL	0.033 pg/mL	[132]
SERS	CA19-9	R6G-tagged MoS_2_ NF	5 × 10^−3^–100 IU/mL	3.43 × 10^−4^ IU/mL	[140]
SERS	exosomes	MoS_2_-AuNSs/ROX-Apt	55–5.5 × 10^5^ particles/μL	17 particles/μL	[131]
SPR	CYFRA21-1	Ab/COOH-MoS_2_/Au/Cr/BK7	0.05 pg/mL–100 ng/mL	0.05 pg/mL	[132]

**Table 3:** Miscellaneous biosensors for cancer biomarkers detection based on MoS_2_.

Method	Analytes	Electrode/Label	Linear Range	LOD	Ref.
FET array	NMP22 & CK8	MoS_2_ NSs-FET	10^−6^–10^−1^ pg/mL	0.027–0.019 aM	[144]
DeMEA/ microfluidic	exosome	GASI microfluidic channel/anti-EpCAM	1 × 10^2^–1 × 10^9^ exosomes/μL	17 exosomes/μL	[150]
EIS/CV	T_3_	Au-MoS_2_/anti-T_3_ electrodes	0.01–100 ng/mL	2.5 pg/mL	[151]
SAW	CEA	AuNP–MoS_2_-rGO/PI	0.1–80 ng/mL.	0.084 ng/mL	[152]
bio-FET	PSA	MoS_2_/B-SA system with DNA tetrahedron	1 fg/mL–100 ng/mL (PBS) 1 fg/mL–100 ng/mL (serum)	1 fg/mL	[145]
Paper-Based/DPV	miRNA-1 55miRNA-21	AuNPs/RGO/PE AuNPs/MoS_2_/PE	33.8–135.3 nM 135.6–406.8 nM	12.0 nM 25.7 nM	[153]
Microfluidic/ electrochemical/visual	miRNA-141	Bi_2_S_3_@MoS_2_ NFs/CeO_2_	10 fM–1 nM 0.5 fM–1 nM	0.12 fM 2.65 fM	[154]
signal amplification/ 3D DNA walker	microRNA-199a	AuNPs/MoS_2_@C	0.0001–100 pM	4.94 amol/L	[142]
SERS/ELISA	ferritin	MoS_2_ @Fe_3_O_4_/BP	10–10^−4^ μg/mL	7.3 × 10^−5^ μg/mL	[155]
SERS/ELISA	CA125	Fe_3_O_4_@MoS_2_/g-C_3_N_4_ NSs	10^−3^–10^2^ IU/mL	4.96 × 10^−4^ IU/mL	[143]
colorimetric/ electrochemical dual mode	miRNA-199a	MoS_2_ NRs/CHA /HCR/MB	0.1–10^5^ fM & 0.1–10^7^ fM	0.0241 aM & 0.0345 aM	[147]
colorimetric/ electrochemical dual mode	HER2	3D DNA walker /1T-MoS_2_/graphdiyne	0.0001–10 pg/mL	0.03 pg/mL	[148]
capacitance electrobiosensor	exosome	MoS_2_ heterolayer/ IDMGE	10^4^–10^8^ exosomes/mL	2192.6 exosomes/mL	[149]
ECL/LSPR	miRNA-210	S dots/AuNPs@MoS_2_ NSs heterostructure	0.1 pM–10 nM	0.03 pM	[136]
electrochemistry	PSA	MoS_2_/SiO_2_ NPs	1 fg/mL–500 ng/mL	2.5 fg/mL	[156]
ECL/CHA	miRNA-210	MoS_2_ NSs@biomimetic magnetic vesicles	1 fM–100 pM	0.3 fM	[157]
LSAW aptasensor	AFP	monolayer MoS_2_/Au NPs/aaptamer	0.01–100 ng/mL	4.79 pg/mL	[158]
PEC/EIS	miR-92a-3p	MoS_2_@Ti_3_C_2_	1–10^8^ fM	0.27 fM	[159]
EIS/microscopic	OFA/iLRP	AB3/GO/SPE	10–300 k cells/mL	16 cells/mL	[160]
FET	BRCA1	Ag-Au@Ins/Apta-MIP without template modified Au gate MoS_2_ FET	10 aM–1.0 nM	6.4 aM	[161]
Fluorescence/ ECL	miRNA-21 miRNA-135b	carbon dots MoS_2_ QDs	0.1 pM–1 nM 30 fM–20 nM	21.1 fM 10 fM	[162]

## Data Availability

Not applicable.

## Abbreviations

AuNPs	gold nanoparticles;
rGO	reduced graphene oxide;
PDDA	poly dimethyl diallyl ammonium chloride;
Ag/AgCl	silver/silver chloride;
ITO	indium tin oxide;
AMACR	alpha-methylacyl-CoA racemase;
NaBH_4_	sodium borohydride;
4-NP	4-nitrophenol;
ssDNA	single strand DNA;
AgNCs	gold nanocubes;
AuNS	gold nanostars;
CTC	circulating tumor cells;
PD-1	programmed cell death protein 1;
CYFRA21-1	cytokeratin 19 fragment (1);
T_3_	triiodothyronine;
DSN	duplex-specific nuclease.
LSAW	Love-mode surface acoustic wave
OFA/iLRP	Oncofetal antigen/immature laminin receptor protein
BRCA1	Breast cancer susceptibility gene type 1
